# LOH at 6q and 10q in fractionated circulating DNA of ovarian cancer patients is predictive for tumor cell spread and overall survival

**DOI:** 10.1186/1471-2407-12-325

**Published:** 2012-07-31

**Authors:** Jan Dominik Kuhlmann, Heidi Schwarzenbach, Pauline Wimberger, Micaela Poetsch, Rainer Kimmig, Sabine Kasimir-Bauer

**Affiliations:** 1 Department of Gynecology and Obstetrics, University Hospital of Essen, Hufelandstrasse 55, Essen, D-45122, Germany; 2 Institute of Tumor Biology, University Medical Center Hamburg-Eppendorf, Hamburg, Germany; 3 Institute of Legal Medicine, University Hospital of Essen, Essen, Germany

**Keywords:** Circulating DNA, Loss of heterozygosity, Disseminated tumor cells, DNA-fractionation, Tumor suppressor genes

## Abstract

**Background:**

We recently showed that LOH proximal to *M6P/IGF2R* locus (*D6S1581*) in primary ovarian tumors is predictive for the presence of disseminated tumor cells (DTC) in the bone marrow (BM). For therapy-monitoring, it would be highly desirable to establish a blood-based biomarker. Therefore, we quantified circulating DNA (cirDNA) in sera of 63 ovarian cancer patients before surgery and after chemotherapy, measured incidence of LOH at four cancer-relevant chromosomal loci, correlated LOH with tumor cell spread to the BM and evaluated prognostic significance of LOH.

**Methods:**

cirDNA was fractionated into high- and low molecular-weight fraction (HMWF, LMWF) for LOH-profiling, utilizing PCR-based fluorescence microsatellite analysis. BM aspirates were analyzed for DTC by immunocytochemistry using the pan-cytokeratin antibody A45-B/B3.

**Results:**

cirDNA levels in the HMWF before surgery were predictive for residual tumor load (p = 0.017). After chemotherapy, we observed a significant decline of cirDNA in the LMWF (p = 0.0001) but not in the HMWF. LOH was prevalently detected in the LMWF with an overall frequency of 67%, only moderately ablating after chemotherapy (45%). Before surgery, LOH in the LMWF at marker *D10S1765* and *D13S218* significantly correlated with tumor grading and FIGO stage (p = 0.033, p = 0.004, respectively). In both combined fractions, LOH at *D6S1581* additionally associated with overall survival (OS) (p = 0.030). Moreover, solely LOH at *D10S1765* in LMWF after therapy correlated with DTC in BM after therapy (p = 0.017).

**Conclusion:**

We demonstrate the applicability and necessity of DNA-fractionation prior to analyzing circulating LOH and identify LOH at *D10S1765* and *D6S1581* as novel blood-based biomarkers for ovarian cancer, being relevant for therapy-monitoring.

## Background

Epithelial ovarian cancer accounts for most tumor-related deaths among female malignancies [1]. At the time of primary diagnosis, 70% of the ovarian cancer patients have advanced tumor stages. Although there are high response rates to carboplatin–based chemotherapy, most of the patients suffer from recurrent disease. Standard treatment of advanced ovarian cancer is primary surgery aiming at macroscopic complete tumor resection and subsequent platinum- and paclitaxel-based chemotherapy [2]. Residual postoperative tumor burden is one of the most important prognostic factors in ovarian cancer [3]. However, despite advances in treatment strategies, more than 50% of all patients will experience recurrence [4], resulting in worse prognosis. Innovative therapy approaches are therefore needed to improve the patient’s outcome.

Thus, the detection and characterisation of new biomarkers is of high interest. We previously analyzed primary ovarian tumors for loss of heterozygosity (LOH) incidence at a panel of four microsatellite markers associated with ovarian cancer-relevant tumor suppressor genes involved in apoptosis, platinum sensitivity and DNA-repair, namely *PTEN* (marker *D10S1765*), *BRCA1* (marker *D13S218*), *BRCA2* (marker *D17S855*) and *M6P/IGF2R* (*D6S1581*). We revealed that LOH incidence in the primary tumor at marker *D6S1581* (directed proximal to *M6P/IGF2R* locus) is predictive for the presence of disseminated tumor cells (DTC) in the bone marrow (BM) before surgery and after chemotherapy [5]. However, primary tumor tissue is only available by resection, and it would be highly desirable to establish a blood-based biomarker which is suitable to monitor the course of disease. In this regard, cell-free nucleic acids, detectable in the blood [6,7], could serve as a tool to detect and characterise residual tumor load [8]. It was denoted, that cancer patients harbor higher concentrations of cirDNA in their blood than normal healthy donors [7] and already in the 1980s, it was suggested that cirDNA in the circulation of cancer patients might originate from malignant cells [9,10]. Up to now, a variety of tumor specific alterations like T790M *EGFR* mutations in lung cancer [11] could be detected in cirDNA of cancer patients. Previous studies on allelic loss in serum of cancer patients usually analyzed non-fractionated cirDNA, which is largely diluted by contaminating normal DNA and thus, a broad range of LOH detection rates with partly contradictory results was observed [12-14]. In a recently published study on prostate cancer, we could technically improve sensitivity of LOH detection in cirDNA by a sequential purification procedure with two different column systems in order to fractionate cirDNA into high-molecular-weight fraction (HMWF) and low-molecular-weight fraction (LMWF) [15]. However, for ovarian cancer, no data on circulating allelic loss exist so far. Therefore, in the present study, we intended to extent our previous LOH investigation from the primary tumor to the patient’s blood sera obtained at primary diagnosis and after chemotherapy, utilizing a DNA fractionation technique [15]. The purpose was to monitor levels of cirDNA, to describe incidence and pattern of LOH at four ovarian cancer-relevant chromosomal loci, to correlate LOH occurrence with tumor cell spread to the BM and finally to evaluate prognostic significance of LOH in the blood of ovarian cancer patients.

## Methods

### Characterisation of study patients

The present study was conducted at the Department of Gynecology and Obstetrics at the University Hospital in Essen. Patients with primary epithelial ovarian cancer were enrolled from February 2001 until November 2007. In total, sera of 63 ovarian cancer patients and sera of 20 healthy donors were studied. Overall survival (OS) data of these patients were obtained from the local municipal registry. The median follow-up time was 3.04 years, ranging from 0.08 to 5.83 years. Informed written consent was obtained from all patients, and the study was approved by the Local Essen Research Ethics Committee (05/2856). Clinical data of the patients are summarized in Table 1. Radical tumor debulking was performed when feasible. Radical pelvic and para-aortic lymphadenectomy was performed, if macroscopic complete tumor resection was achieved. Chemotherapy consisted of six cycles of carboplatinum (AUC 5) and paclitaxel (175 mg/m^2^). Grading was performed according to WHO classification. Patients who had a treatment-free interval of 0–5 months after first-line chemotherapy can appropriately be considered to have clinically defined platinum resistant disease.

**Table 1:** Patient Characteristics at the Time of Primary Diagnosis of Ovarian Cancer

**Patients (%)**	
**Total**	63
**Age**	58 years (range 21 – 81 years)
**Tumor stage**	
FIGO I-II	13 (20.6)
FIGO III	35 (55.6)
FIGO IV	15 (23.8)
**Lymph node metastasis**	
N_0_	22 (34.9)
N_1_	20 (31.7)
N_x_	21 (33.3)
**Tumor grading**	
I - II	31 (49.2)
III - IV	32 (50.8)
**Histologic subtype**	
serous papillary	
subtype	50 (79.4)
other subtypes	13 (20.6)
**Residual tumor**	
macroscopic	
complete resection	30 (47.6)
any residual tumor	33 (52.4)
**Survival**	
OS^a^	36.5 months (range 1 – 70 months)
Alive	28 (44.4)
Dead	34 (54.0)
DFS^b^	12 months (range 6 – 48 months)
no relapse	18 (28.6)
relapse	45 (71.4)
**Platinum Resistance**	
Platinum sensitive 54 (85.7)	
Platinum resistant	9 (14.3)
**DTC**^c^	
DTC prior to chemotherapy	
Positive	27 (50.9)
Negative	26 (49.1)
DTC after chemotherapy	
positive	20 (50.0)
negative	20 (50.0)

^a^OS, overall survival.

^b^DFS, disease-free survival.

^c^DTC, disseminated tumor cells in BM.

### Preparation of blood serum

Nine ml blood were drawn from each patient, stored at 4°C and processed within 4 h to avoid blood cell lysis. Blood fractionation was carried out by centrifugation for 10 min at 2500 g. Subsequently, 3 – 4 ml of the upper phase, constituting blood serum, were removed and subjected to isolation of cirDNA.

### DNA extraction and fractionation

As described previously [15], DNA of the HMWF was extracted using the QIAamp DNA Mini Kit (Qiagen, Hilden, Germany). Subsequently, the flow-through from the Qiagen columns was mixed with 2 volumes of 6 M guanidine thiocyanate and purified on Wizard Plus columns (Promega, Mannheim, Germany) to obtain the LMWF. Genomic DNA from matching paraffin embedded tumor-free lymph nodes (reference DNA) was extracted using the QIAamp Blood DNA Mini Kit (Qiagen). Quantity and quality of the extracted DNA were determined spectrophotometrically using the NanoDrop Spectrometer ND-1000 (NanoDrop, Delaware, USA). All steps were performed according to the manufacturer’s instructions.

### Fluorescence-labeled PCR

DNA was selectively amplified by PCR reaction using primer pairs binding to microsatellite markers of interest, previously described by us in detail [5]. PCR conditions were: 10 ng of DNA were amplified in a 10-μl-reaction containing PCR Gold buffer (150 mM Tris–HCl, pH 8.0 and 500 mM KCl), 2.5 mM MgCl_2_ (Applied Biosystems, Mannheim, Germany), 200 μM dNTPs (Roche, Mannheim, Germany), 0.2 μM of primer sets (Sigma, Taufkirchen, Germany) and 0.5 U AmpliTaq Gold DNA-Polymerase (Applied Biosystems). The sense primers were fluorescence-labeled (HEX, FAM or TAMRA) at the 5' end. The reaction was initialised by heat-activation of the DNA polymerase (10 min at 95°C) followed by 40 cycles of PCR amplification. Tetramethylammonium chloride (TMAC) at a final concentration of 0.1 mM was added to the PCR reaction. As previously shown, the addition of TMAC increases the yield of PCR products, enhances specificity and decreases the probability of primer dimerisation, formation of slippage peaks and artificial LOH [15-17].

### Evaluation of PCR products

The fluorescence-labeled PCR products were separated by capillary gel electrophoresis and detected on an automated Genetic Analyzer 310 (Applied Biosystems, Germany). Fragment length and fluorescence intensity were evaluated by the GeneScan software. The 500-ROX size marker (Applied Biosystems) served as an internal standard. LOH incidence was determined by calculating the ratio of intensities of the two alleles from a tumor sample corrected by that from the corresponding tumor-free lymph node sample (reference DNA). Shift in allelic ratio was interpreted as LOH if the final quotient was ≤ 0.6 or > 1.67. All experiments were performed in duplicates, homozygous and non-analyzable peaks were designated as non-informative cases. Previously, dilution experiments were performed to calculate the lowest portion of tumor-specific DNA that could be detected. For this purpose, known proportions of normal DNA (derived from leukocytes) and tumor DNA were mixed and amplified [15]. All experiments were performed in triplicates. LOH positivity for a respective sample was exclusively considered valid in case of two independently obtained LOH-positive results. Homozygous and non-analyzable peaks as well as sample triplets with any further indication for amplification abnormality (e.g. strong intra-triplicate peak-ratio fluctuation or low fluorescence level) were designated as non-informative.

### Collection and analysis of BM

BM was bilaterally aspirated from the anterior iliac crests of 53 primary ovarian cancer patients before surgery and of 40 patients after chemotherapy (local anaesthesia with mepivacain), respectively and processed within 24 hours. Tumor cell isolation and detection were performed based on the recommendations for standardized tumor cell detection published by the German Consensus Group of Senology [18]. In total, 8 x 10^6^ mononuclear cells (MNC) per patient were analyzed. The slides were air-dried overnight at room temperature.

### Immunocytochemistry

Staining for CK-positive cells was performed using the murine monoclonal antibody Mab A45-B/B3 (Micromet, Munich Germany), directed against a common epitope of CK polypeptides including the CK heterodimers 8/18 and 8/19. This standard procedure for detection of DTC has been described in detail elsewhere [5,18,19].

### Evaluation of CK + Cells

Microscopic evaluation of the slides was carried out using the ARIOL system (Applied Imaging) according to the International Society for Hematotherapy and Graft Engineering (ISHAGE) evaluation criteria and the DTC consensus [18,19]. These automated scanning microscopes and image analysis systems consist of a slide loader, camera, computer and software for the detection and classification of cells of interest, based on particular colour, intensity, size, pattern and shape.

### Statistical analysis

Statistical analysis was performed using the SPSS software package, version 18.0 (SPSS Inc. Chicago, IL). The chi Square or two-tailed Fischer’s exact test and the univariate binary logistical regression were used to identify associations between LOH patterns in the patient’s sera and clinicopathological parameters of ovarian cancer patients including DTC in the BM. In addition, the Mann–Whitney–U and the Wilcoxon-W tests for non parametric comparison of two independent and dependent variables were used, respectively. Kaplan-Meier plots were used to estimate OS and recurrence. The log rank test was used for statistical analysis. A p-value ≤ 0.05 was considered statistically significant. All p-values are two-tailed.

## Results

### Quantification of cirDNA in blood serum of ovarian cancer patients and its clinical relevance

**Figure 1:**
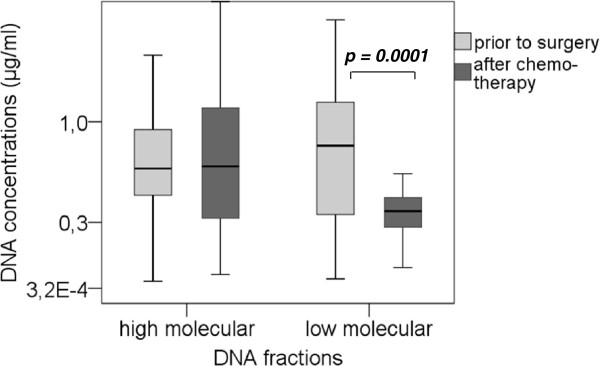
**Quantification of fractionated serum DNA derived from ovarian cancer patients.** The box plot shows the results of spectrophotometrical quantification of cirDNA in the HMWF and the LMWF derived from blood serum of ovarian cancer patients before surgery and after chemotherapy. Statistical significance according to the Mann–Whitney-U test for the non-parametric comparison of two independent variables is indicated.

From a total number of 63 patients before surgery, 58 sera after chemotherapy were available and subjected to isolation of cirDNA, which was fractionated into HMWF and LMWF. In the HMWF, before surgery, a median DNA concentration of 640 ng/ml of serum (range 30 – 2460 ng/ml) was recorded, whereas after chemotherapy, a median DNA content of 660 ng/ml (range 60 – 2820 ng/ml) could be detected (Figure 1). Serum DNA concentrations before surgery and after chemotherapy were similar, albeit the concentration after chemotherapy had an increased distribution. In the LMWF, before surgery, a median value of 810 ng/ml (range 40 – 2060 ng/ml) was detected. After chemotherapy, we observed a highly significant decline of DNA content in the LMWF to a median value of 380 ng/ml (range 40 – 1790 ng/ml) (p = 0.0001). Using the Mann–Whitney-*U* test, statistical evaluation showed that DNA concentration in the HMWF before therapy significantly associated with residual tumor load left after surgery (p = 0.017).

### LOH frequency and distribution in blood serum of ovarian cancer patients

**Figure 2:**
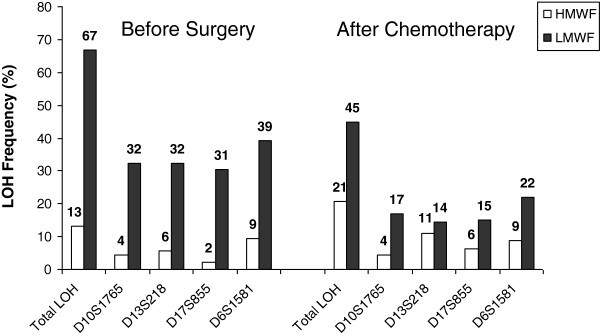
**LOH incidence in blood serum at ovarian cancer-related chromosomal regions.** The bar chart graphically demonstrates the LOH frequencies observed in the HMWF- and LMWF-DNA of blood serum from ovarian cancer patients at four selected microsatellite markers.

All sera of ovarian cancer patients, obtained before surgery and after chemotherapy, were tested for LOH at four ovarian cancer-relevant microsatellite markers, previously described by us in detail [5]. Before Surgery, 31/63 patients (49%) showed at least one LOH in one of the two fractions, whereas after chemotherapy in 24/58 patients (41%), at least one LOH was detectable. The presence of LOH could not be observed in cirDNA of healthy donors (data not shown). Frequency and distribution of LOH incidence are graphically depicted in Figure 2. In the HMWF, LOH was a rare event with an overall detection rate of 13% before surgery and 21% after chemotherapy, whereas in the LMWF, LOH occurred more frequently with an overall detection rate of 67% before surgery and 45% after chemotherapy. Allelic loss at *D6S1581* before surgery and after chemotherapy was most prevalent in both cirDNA fractions and generally, LOH incidence in the LMWF at all markers tended to moderately decrease after chemotherapy. By comparing the incidence of LOH at all four markers in both cirDNA fractions before surgery and after chemotherapy, we denoted that in 25/63 patients (40%), LOH for at least one marker was not detectable anymore after chemotherapy. In 6/63 patients (10%), LOH for at least one marker was concordantly detected before surgery and after chemotherapy, whereas “de novo” occurrence of LOH after chemotherapy was observed in 19/63 patients (30%) (data not shown). 

### Correlation of LOH in blood serum with the presence of DTC in the BM

In total, DTC were studied in 53/63 patients before surgery and in 40/58 patients after chemotherapy. Prior to surgery, DTC were detected in 26/53 patients (49%) with a median number of 6 cells per 8 x 10^6^ MNC (range 1–38 cells). After chemotherapy, 20/40 patients (50%) revealed DTC in their BM with a median number of 10 cells per 8 x 10^6^ MNC (range 1–35 cells). LOH incidence at marker *D10S1765* in HMWF after chemotherapy significantly correlated with the incidence of DTC after therapy (p = 0.017) ( Additional file 1: Table S1).

### Clinical significance of LOH in blood serum

Correlation of LOH incidence in cirDNA with common clinicopathologic parameters was performed ( Additional file 1: Table S1). Before surgery, LOH at marker *D10S1765* in HMWF and *D13S218* in LMWF and in both combined fractions significantly correlated with FIGO stage (p = 0.035, p = 0.033, p = 0.012, respectively). Moreover, LOH at chromosomal site *D10S1765* in LMWF and in both combined fractions correlated with tumor grading (p = 0.004, p = 0.012, respectively). However, no association of LOH with platinum resistance could be observed.

**Figure 3:**
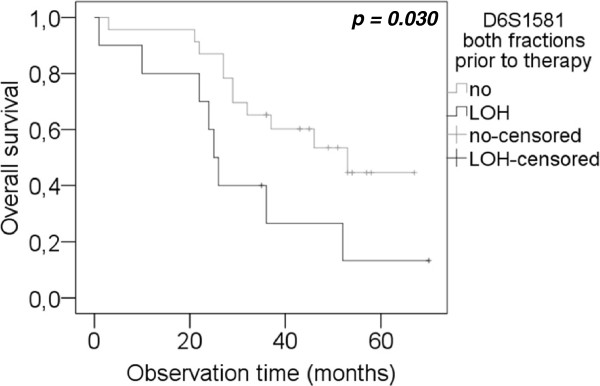
**Correlation of LOH at *D6S1581 *and overall survival.** Kaplan-Meier curves depict overall survival analysis of patients with and without LOH incidence at marker *D6S1581* in a combined analysis of both fractions, before surgery. Top curves, patients with retention of the two alleles at *D6S1581*. Bottom curves, patients with LOH at marker *D6S1581.*

Median disease-free survival (DFS) of the patients was 12 months (range 6 – 48 months) and median OS was 36.5 months (range 1–70 months). As illustrated in Figure 3, LOH at *D6S1581* in both combined fractions was predictive for a reduced OS (p = 0.030). Median OS periods were 25 and 53 months (95% CI 22–28 and 28–78) in patients who displayed LOH and no LOH at *D6S1581* in their serum, respectively. However, no correlation of LOH at the investigated chromosomal regions and DFS of the patients was observed.

## Discussion

In the current study, we show that fractionation of DNA is highly essential for reliable detection of LOH in serum samples of ovarian cancer patients. Applying this new technique, we found more LOH in the LMWF than in the HMWF indicating that tumor-associated DNA is rather short-stranded than long-stranded. This observation is also supported by the fact that after chemotherapy, only the LMWF-DNA levels decreased, whereas the HMWF-DNA levels were similar prior to surgery and after chemotherapy. Moreover, our key findings showed that LOH at *D6S1581* before surgery is predictive for reduced OS (combined fraction analysis) and LOH at *D10S1765* after chemotherapy was significantly associated with tumor cell spread to the BM, respectively.

It has been reported that tumor-associated DNA, released into circulation, is rather of fragmented and low-molecular-weight character [20]. In this context, it was proposed that tumor cells, which proliferate at abnormal rates, frequently undergo apoptosis [21-23] and cumulatively deliver small and fragmented DNA into the circulation. Therefore, it seems reasonable that the observed decline of cirDNA in our patients, selectively observed in the LMWF, can be ascribed to a systematic clearance of tumor cells upon chemotherapy, which leads to a global reduction of low molecular weight DNA. However, we did not see any significant change of HMWF-DNA prior to surgery and after chemotherapy. Long-stranded HMWF-DNA could contain an excess of normal DNA in the circulation and derives from normal blood lymphocytes [24,25], or necrotic stromal and inflammatory cells within the tumor microenvironment [26]. Accordingly, the observed steady-state HMWF-DNA content before surgery and after chemotherapy might indicate that chemotherapy does not sustainably affect HMWF-DNA in blood of ovarian cancer patients.

The majority of allelic losses were detected in the LMWF and also significantly correlated with common clinicopathological parameters of the patients whereas in the HMWF, LOH constituted a comparatively rare event. So far, previous studies on several tumor entities exclusively analyzed non-fractionated cirDNA and thus, there is a broad variety of LOH detection rates with partly contradictory results [12,24]. In cooperation with the laboratory of Schwarzenbach et al., we engaged its newly developed fractionation technique and could show that our findings were in accordance with data on prostate cancer, which showed a higher LOH detection rate in the LMWF compared to the HMWF [15]. To date, with the exception of this study, there is no further investigation of LOH on fractionated cell-free DNA. However, Wang et al. used a column-based, modified Guanidine/Promega Resin (G/R) method to selectively isolate fragmented DNA and showed for colorectal cancer patients that preferential isolation of fragmented DNA enhances the detection of circulating mutated *K-RAS* DNA sequences [20]. In this context, Wang proposed that in order to enhance the detection rate of somatic mutations or epigenetic modifications in circulating blood DNA, a method selectively extracting small DNA fragments should be used. Given these considerations and comprising our data, we can conclude for ovarian cancer that tumor DNA is rather reflected in the LMWF.

In comparison to preoperative sera, LOH incidence only moderately decreased after chemotherapy. Tracing microsatellite alterations in follow-up sera of patients with different cancer entities and investigating their clinical significance has previously been described in several studies [27,28]. In a previous investigation on breast cancer, overall LOH pattern of serial serum DNA samples was largely stable but it remained unclear if the remaining non concordant results were due to changes in clinical status [29]. In another study, the persistence of microsatellite alterations in plasma DNA of post-mastectomy patients has been associated with bad-prognosis histological parameters and indicated micrometastatic disease [30]. The detection of tumor associated microsatellite alteration after accomplished chemotherapy could indicate the presence of residual primary tumor or occult micrometastatic cells, such as circulating tumor cells or DTC, being resistant to treatment.

Apart from patients who’s LOH vanished upon chemotherapeutic intervention, a subset of patients displayed LOH events after chemotherapy which were not detectable before surgery. This observation has also been described in small cell lung cancer patients (SCLC). In case of a successful therapy, molecular alterations on cell-free DNA concomitantly vanished and new LOH events after chemotherapy coincided with disease recurrence [31]. A statistically substantiated conclusion about the prognostical impact of “de novo” LOH occurrence after chemotherapy in our patient cohort cannot be performed based on the limited number of this patient group. However, one can conceptually speculate that a certain subset of cancer cells with stem like properties is not affected by cytoreductive treatment, might evolve by clonal evolution, might systematically acquire de novo genetic alterations and might become capable of effecting recurrence. Accordingly, it could be observed previously that a side population of tumor cells with stem cell features, overexpressing ABC drug transporters, sustained the growth of drug-resistant ovarian tumors and induced recurrence [32].

Considering LOH at the different loci, we observed that LOH at *D10S1765* in LMWF after therapy was predictive for tumor cell dissemination to BM after therapy, FIGO stage and tumor grading. Significant associations of this marker with FIGO stage and grading coincide with our previous LOH investigation in primary ovarian tumor tissue [5]. *D10S1765* is located at chromosomal band 10q23.3. This region encodes for PTEN, a dual-specificity protein phosphatase antagonizing the PI3K-Akt signaling pathway and regulating cellular proliferation, DNA repair, stem cell self-renewal, genomic instability and metastasis [33]. Particularly, in ovarian cancer, PTEN down-regulation has been shown to be functionally involved in platinum resistance [34]. It seems rational that highly advanced tumors are more dependent on the accumulation of genetic alterations than early-stage neoplastic lesions. Furthermore, it can be hypothesized that *PTEN* locus disruption by LOH, in combination with other inactivation mechanisms such as epigenetic events [35], might functionally be involved in tumor progression, persistence of tumor cells after chemotherapy and their capacity to disseminate to the BM.

We revealed that LOH in cirDNA of ovarian cancer patients is predictive for FIGO stage and tumor grading. Moreover, one of our key finding was that LOH at marker *D6S1581* in a combined analysis of both fractions, detected at primary diagnosis, was predictive for reduced OS. Microsatellite marker *D6S1581* is located at 6q25.1, 25 kb proximal to the mannose 6-phosphate/insulin-like growth factor II receptor (*M6P/IGF2R*) locus [36]. This receptor is well described in breast cancer [37-39] and is supposed to act as a tumor suppressor gene by negatively regulating cell survival, tumor invasion and metastasis [40]. Moreover, LOH at *M6P/IGF2R* is well known to be a frequent event in primary ovarian cancers [41] and a low *D6S1581* copy number was associated with platinum-resistant condition in ovarian cancer [36]. However, in our patient cohort, LOH occurrence did not significantly associate with platinum resistance. Generally, advanced ovarian cancer is a relatively chemo-sensitive tumor with overall clinical response rates of 70 – 80% [42]. Therefore, the observed number of resistant cases in our study (14.3%) might be too limited to allow a reliable and statistical substantiated conclusion.

The ascertained prognostic impact of marker *D6S1581* in cirDNA of ovarian cancer patients in our current study is of particular interest, as our present serum data corroborate our previous findings on ovarian tumor tissue showing that allelic loss at *D6S1581* is a biomarker for tumor cell spread to the BM [5]. Thus, evidence is accumulating, that genomic region proximal to *M6P/IGF2R* locus seems to be functionally implicated for ovarian tumorigenesis and may serve as a blood-based biomarker for prognosis. These data clearly sustain the reliability of cell-free cirDNA as “real-time liquid biopsy”, providing valuable prognostic information and being accessible non-invasively. Moreover, most of the significant associations with the clinicopathological parameters were found at the time of primary diagnosis before surgery. Thus, preoperative serum seems to constitute most informative reading point for patients with primary ovarian cancer.

The occurrence of artificial LOH in the LMWF, which is supposed to be random allelic loss due to low DNA concentration or impaired DNA integrity, constitutes a possible draw-back in the context of low-molecular-weight DNA amplification. However, random allelic loss due to a suboptimal DNA integrity would logically be accompanied by a statistically more frequent loss of the “shorter” allele of a polymorphic microsatellite maker, which should be more difficult to amplify than the “longer” allele. Despite from the addition of TMAC in order to prevent artificial LOH and to improve amplification condition, we found the number of losses of “short” and “long” alleles being well balanced throughout the entire marker panel and throughout all patients. Thus, it is very unlikely that observed LOH in the LMWF reflect a random process.

We previously tried to characterize fragment size distribution of HMWF and LMWF [15], but, however, due to our experimental conditions and due to a partly overlapping performances of the different filter-column systems, so far, it was not possible to narrow down an exact cut-off for cirDNA fractionation. In this context, further studies are planned to better characterize size distribution of cirDNA fractions.

Conclusively, in the present study, we demonstrate the applicability and necessity of DNA fractionation in order to selectively monitor tumor associated microsatellite alterations in blood serum of ovarian cancer patients. Given our present findings, tumor DNA seems to be mostly reflected in the LMWF. It is of note that tumor specific circulating LOH with diagnostic relevance is not exclusively limited to the LMWF. Based on our findings, we have to consider that the HMWF might also contain a certain proportion of tumor derived DNA and should additionally be analyzed for blood-based LOH studies on ovarian cancer. Moreover, we newly identified LOH at *D10S1765* and *D6S1581* as novel blood-based biomarkers for tumor cell spread to the BM and prognosis, respectively, which might be of clinical relevance for monitoring studies. Genomic region proximal to *M6P/IGF2R*, a promising target, and its role in ovarian cancer is currently under investigation.

## Conclusion

We demonstrate the necessity and applicability of DNA-fractionation prior to analyzing circulating allelic loss in the blood of ovarian cancer patients. Moreover, we identified novel circulating biomarkers and sustain diagnostic impact of cirDNA in terms of a “real-time liquid biopsy”.

## Abbreviations

LOH: Loss of heterozygosity; BM: Bone marrow; cirDNA: Circulating DNA; HMWF: High-molecular-weight fraction; LMWF: Low-molecular-weight fraction; OS: Overall survival; TMAC: Tetramethylammonium chloride; MNC: Mononuclear cells; CK: Cytokeratin; ISHAGE: International Society for Hematotherapy and Graft Engineering; ROC-curve: Receiver operating characteristics curve; AUC: Area under the curve; DFS: Disease-free survival; MNC: Mononuclear cells; CI: Confidence interval; G/R method: Guanidine/Promega-Resin method.

## Competing interests

The authors declare that they have no competing interests.

## Authors’ contributions

JDK, HS, MP, SKB made substantial contributions to the conception and design of the study, acquisition of data, and analysis and interpretation of the data. JDK, PW, HS, RK and SKB were involved in drafting the manuscript or revising it. All authors read and approved the final manuscript.

## Pre-publication history

The pre-publication history for this paper can be accessed here:

http://www.biomedcentral.com/1471-2407/12/325/prepub

## Supplementary Material

Additional file 1 Table S1. Correlations of LOH Incidence in Blood of Ovarian Cancer Patients in Relation to Clinicopathological Parameters. Click here for file
