# Supplementary material for: LOH at 6q and 10q in fractionated circulating DNA of ovarian cancer patients is predictive for tumor cell spread and overall survival
Source: BMC Cancer. 2012 Jul 31;12:325. doi: 10.1186/1471-2407-12-325 (PMC3488536; doi:10.1186/1471-2407-12-325)
Supplement: Additional file 1 — Table S1. Correlations of LOH Incidence in Blood of Ovarian Cancer Patients in Relation to Clinicopathological Parameters. [file 1471-2407-12-325-S1.doc]

**Supplementary Table 1:** Correlations of LOH Incidence in Blood of Ovarian Cancer Patients in Relation to Clinicopathological Parameters

|  | **DNA concentration**  HMWF^b^  before surgery | LOH ***D10S1765***  HMWF **and** LMWF^d^  before surgery | LOH ***D10S1765***  HMWF  before surgery | LOH ***D10S1765***  LMWF  before surgery | LOH ***D10S1765***  LMWF  after chemotherapy | LOH  ***D13S218***  LMWF  before surgery | LOH  ***D13S218***  HMWF **and** LMWF  before surgery | LOH  ***D6S1581***  HMWF **and** LMWF  before surgery |
| --- | --- | --- | --- | --- | --- | --- | --- | --- |
| FIGO | n.s.^c^ | n.s. | **p = 0.035** | n.s. | n.s. | **p = 0.033** | **p = 0.012** | n.s. |
| Nodal status | n.s. | n.s. | n.s. | n.s. | n.s. | n.s. | n.s. | n.s. |
| Metastasis  (FIGO IV) | n.s. | n.s. | n.s. | n.s. | n.s. | n.s. | n.s. | n.s. |
| Tumor grading | n.s. | **p = 0.012** | n.s. | **p = 0.004** | n.s. | n.s. | n.s. | n.s. |
| Residual tumor load | **p = 0.017** | n.s. | n.s. | n.s. | n.s. | n.s. | n.s. | n.s. |
| Histology | n.s. | n.s. | n.s. | n.s. | n.s. | n.s. | n.s. | n.s. |
| Platinum resistance | n.s. | n.s. | n.s. | n.s. | n.s. | n.s. | n.s. | n.s. |
| DFS/PFS | n.s. | n.s. | n.s. | n.s. | n.s. | n.s. | n.s. | n.s. |
| OS | n.s. | n.s. | n.s. | n.s. | n.s. | n.s. | n.s. | **p = 0.030** |
| DTC^a^  after chemotherapy | n.s. | n.s. | n.s. | n.s. | **p = 0.017** | n.s. | n.s. | n.s. |

^a^*DTC*, disseminated tumor cells in the bone marrow

^b^*HMWF*, high-molecular-weight fraction of circulating DNA

^c^*n.s.*, no significant correlation observed

^d^*LMWF*, low-molecular-weight fraction of circulating DNA
